# Vaccinia Virus BBK E3 Ligase Adaptor A55 Targets Importin-Dependent NF-κB Activation and Inhibits CD8^+^ T-Cell Memory

**DOI:** 10.1128/JVI.00051-19

**Published:** 2019-05-01

**Authors:** Mitchell A. Pallett, Hongwei Ren, Rui-Yao Zhang, Simon R. Scutts, Laura Gonzalez, Zihan Zhu, Carlos Maluquer de Motes, Geoffrey L. Smith

**Affiliations:** a Division of Virology, Department of Pathology, University of Cambridge, Cambridge, United Kingdom; University of Illinois at Urbana Champaign

**Keywords:** BTB-Kelch, E3 ligase, NF-κB, cullin-3, importins, protein A55, vaccinia virus

## Abstract

Viral infection of cells is sensed by pathogen recognition receptors that trigger an antiviral innate immune response, and consequently viruses have evolved countermeasures. Vaccinia virus (VACV) evades the host immune response by expressing scores of immunomodulatory proteins. One family of VACV proteins are the BTB-BACK (broad-complex, tram-trac, and bric-a-brac [BTB] and C-terminal Kelch [BACK]) domain-containing, Kelch-like (BBK) family of predicted cullin-3 E3 ligase adaptors: A55, C2, and F3. Previous studies demonstrated that gene *A55R* encodes a protein that is nonessential for VACV replication yet affects viral virulence *in vivo*. Here, we report that A55 is an NF-κB inhibitor acting downstream of IκBα degradation, preventing gene transcription and cytokine secretion in response to cytokine stimulation. A55 targets the host importin α1 (KPNA2), acting to reduce p65 binding and its nuclear translocation. Interestingly, while A55 was confirmed to coprecipitate with cullin-3 in a BTB-dependent manner, its NF-κB inhibitory activity mapped to the Kelch domain, which alone is sufficient to coprecipitate with KPNA2 and inhibit NF-κB signaling. Intradermal infection of mice with a virus lacking *A55R* (vΔA55) increased VACV-specific CD8^+^ T-cell proliferation, activation, and cytotoxicity in comparison to levels of the wild-type (WT) virus. Furthermore, immunization with vΔA55 induced increased protection to intranasal VACV challenge compared to the level with control viruses. In summary, this report describes the first target of a poxvirus-encoded BBK protein and a novel mechanism for DNA virus immune evasion, resulting in increased CD8^+^ T-cell memory and a more immunogenic vaccine.

**IMPORTANCE** NF-κB is a critical transcription factor in the innate immune response to infection and in shaping adaptive immunity. The identification of host and virus proteins that modulate the induction of immunological memory is important for improving virus-based vaccine design and efficacy. In viruses, the expression of BTB-BACK Kelch-like (BBK) proteins is restricted to poxviruses and conserved within them, indicating the importance of these proteins for these medically important viruses. Using vaccinia virus (VACV), the smallpox vaccine, we report that the VACV BBK protein A55 dysregulates NF-κB signaling by disrupting the p65-importin interaction, thus preventing NF-κB translocation and blocking NF-κB-dependent gene transcription. Infection with VACV lacking A55 induces increased VACV-specific CD8^+^ T-cell memory and better protection against VACV challenge. Studying viral immunomodulators therefore expands not only our understanding of viral pathogenesis and immune evasion strategies but also of the immune signaling cascades controlling antiviral immunity and the development of immune memory.

## Abstract (precis)

NF-κB is a critical transcription factor in the innate immune response to infection and in shaping adaptive immunity. The identification of host and virus proteins that modulate the induction of immunological memory is important for improving virus-based vaccine design and efficacy. In viruses, the expression of BTB-BACK Kelch-like (BBK) proteins is restricted to poxviruses and conserved within them, indicating the importance of these proteins for these medically important viruses. Using vaccinia virus (VACV), the smallpox vaccine, we report that the VACV BBK protein A55 dysregulates NF-κB signaling by disrupting the p65-importin interaction, thus preventing NF-κB translocation and blocking NF-κB-dependent gene transcription. Infection with VACV lacking A55 induces increased VACV-specific CD8^+^ T-cell memory and better protection against VACV challenge. Studying viral immunomodulators therefore expands not only our understanding of viral pathogenesis and immune evasion strategies but also of the immune signaling cascades controlling antiviral immunity and the development of immune memory.

## INTRODUCTION

Virus infection is sensed by pattern recognition receptors (PRRs) that detect pathogen-associated molecular patterns (PAMPs), and this triggers an innate immune response to restrict viral replication and spread. PRR ligand binding leads to an intracellular phosphokinase signaling cascade(s) and activation of specific inflammatory transcription factors, including nuclear factor kappa-light chain-enhancer of activated B cells (NF-κB), interferon regulatory factor 3, 7, or 9 (IRF3/7/9), and signal transducer and activator of transcription (STAT) proteins (1). Activation of these transcription factors controls the host innate immune response by the direct and specific transcriptional regulation of proinflammatory and antiviral genes. Canonical NF-κB signaling mediated via tumor necrosis factor receptor (TNFR), interleukin-1 receptor (IL-1R), retinoic acid-induced gene 1 (RIG-I)-like receptors (RLRs), and DNA sensing converges at the inhibitor of kappa B kinase (IKK) (1). Phosphorylated IKKβ, in turn, phosphorylates the inhibitor of NF-κB subunit alpha (IκBα) that induces its cullin-1/β-TrCP-dependent degradation (2). Under normal conditions IκBα tethers NF-κB in the cytoplasm, preventing NF-κB-dependent gene transcription. Free NF-κB binds to importin α1 to α4 (karyopherin subunit alpha 1 [KPNA1] to KPNA4)/importin β and translocates into the nucleus (3–6) where, following phosphorylation-dependent CBP/P300 acetylation (7), it upregulates NF-κB-dependent gene transcription. NF-κB-induced cytokines and chemokines then promote inflammation and immune cell recruitment to clear infection.

During their evolution viruses have acquired proteins to counteract the host immune response. A prototypical example is vaccinia virus (VACV), the live vaccine used to eradicate smallpox. VACV is a member of the genus *Orthopoxvirus* of the *Poxviridae*, a family of large double-stranded DNA viruses that replicate in the cytoplasm. To evade the host immune response, VACV encodes a plethora of immunomodulatory proteins, including more than 10 inhibitors of NF-κB activation that function at different stages in the pathway (8). For instance, protein B14 targets IKKβ to prevent IκBα phosphorylation and degradation and subsequent p65 nuclear translocation (9). K7 was reported to coprecipitate with TRAF6 and IRAK2 to suppress Toll-like receptor (TLR)-dependent NF-κB activation (10), and A49 blocks β-TrCP-mediated degradation of phosphorylated IκBα via molecular mimicry (11). Although 10 VACV inhibitors of NF-κB have been described, others exist because a virus lacking all these inhibitors still inhibited NF-κB activation (12). Ectromelia virus, a related orthopoxvirus that causes mousepox, encodes several inhibitors of NF-κB, including the protein EVM150 (13) that shares 93% amino acid identity with the VACV Western Reserve (WR) strain protein A55. A55 belongs to the BBK (broad-complex, tram-trac and bric-a-brac [BTB] and C-terminal Kelch [BACK]) family of proteins (14). Interestingly, outside mammals, only poxviruses encode Kelch-like proteins (15).

BTB-Kelch proteins are substrate-specific adaptors for the cullin-3 ubiquitin-ligase complex and regulate modification and/or degradation of various proteins by ubiquitylation (16). Several orthopoxvirus BBK proteins coprecipitate with cullin-3 (15). The N-terminal BTB domain is essential for cullin-3 interaction and contains a conserved tertiary structure consisting of five α-helices with A1 and A2 (A1/2) and A4/5 forming α-helical hairpins and three β-strands (B1/B2/B3) forming a β-sheet. The B1/B2/A1/A2/B3 region is connected to the A4/A5 region by helix A3 and a variable linker region (17). The Kelch motif is a segment of 44 to 56 amino acids with low overall sequence identity, but eight key conserved residues, including four hydrophobic residues followed by a double glycine element separated from two characteristically spaced aromatic residues, tyrosine and tryptophan (18). Each Kelch motif represents one β-sheet blade, and several (4–7) of these repeats can form a beta-propeller (19) and a substrate binding domain. Traditionally, the Kelch domain acts as the E3 ligase substrate receptor and therefore controls substrate specificity. BBK proteins through variation within their Kelch domains modulate a wide range of cellular processes, including actin association/cytoskeleton organization, cell morphology, innate immunity, and gene expression (18). For example, KLHL20 targets IKKβ to downregulate NF-κB signaling (20), while KLHL12 disrupts Wnt-β-catenin signaling through the recruitment of Dishevelled to the cullin-3 E3 ligase complex and its subsequent ubiquitylation and degradation (21).

VACV encodes three BBK proteins, A55, C2, and F3, and one BTB-only protein, C5 (14). Like many other VACV immunomodulators, the VACV BBKs are expressed early during infection (22) and share low amino acid identity. This is 23.5% between A55 and F3 and 18.7% and 18.3% between C2 and A55 and F3, respectively. Previous studies have demonstrated that the genes *A55R*, *F3L*, and *C2L* encode proteins that are nonessential for virus replication yet affect virulence in an intradermal mouse model (23–25). C2 and F3 modulate immune cell recruitment and proliferation *in vivo* (24, 25). Although the virus lacking the gene *A55R* (vΔA55) has altered virulence, how A55 affects virulence and whether it recruits cullin-3 or inhibits inflammatory signaling remain unknown. Thus, we investigated the effect of A55 on host innate immune signaling pathways *in vitro* and *in vivo* and whether this modulated the immune response *in vivo* and/or made for a more protective vaccine.

## RESULTS

### A55 specifically inhibits NF-κB activation *in vitro*.

**FIG 1:**
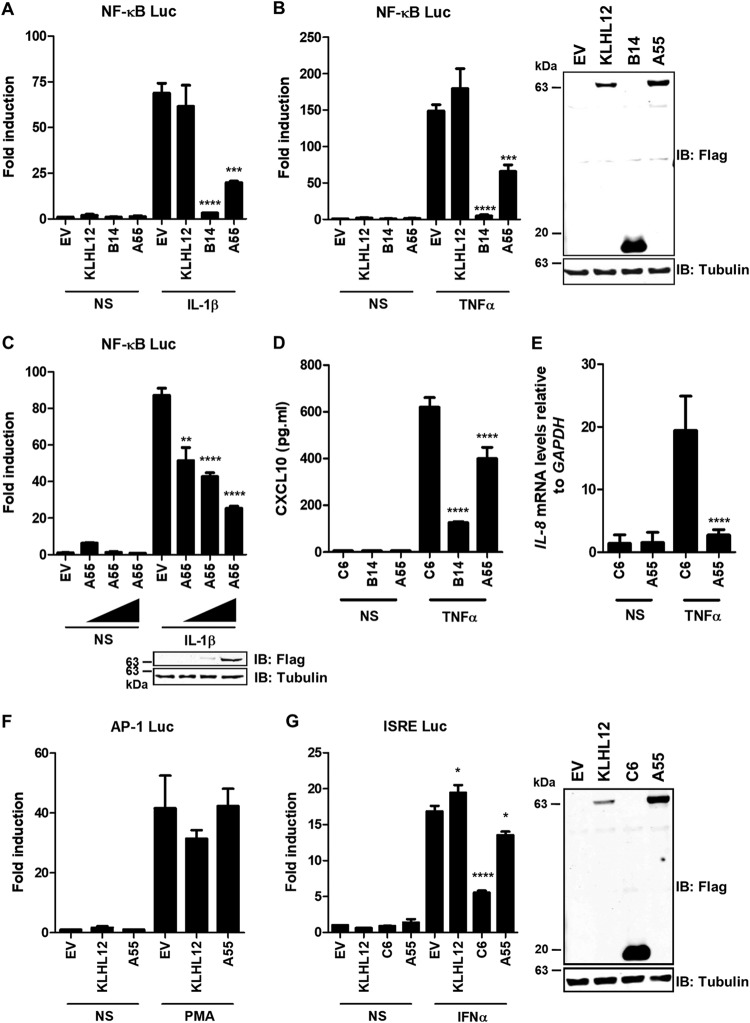
A55 inhibits NF-κB-dependent signaling. (A and B) HEK293T cells were transfected with pLuc-NF-κB and pRL-TK (see Materials and Methods) and plasmids expressing Flag-tagged KLHL12, B14, or A55 or empty vector (EV). After 24 h cells were stimulated with 15 ng/ml IL-1β or 20 ng/ml TNF-α, as indicated, for 6 h. Cell lysates were prepared, and the fold increase in luciferase activity relative to *Renilla* activity was determined. In parallel, cell lysates were analyzed by SDS-PAGE and immunoblotting with anti-Flag or anti-α-tubulin to determine protein expression levels from unstimulated samples. Data are representative of three independent experiments. Statistical significance compares results for the EV-stimulated sample to those of the test sample. (C) The same experiment as described for panel A using increasing plasmid concentrations of pCNDA4/TO-nTAP A55 at 25, 75, and 150 ng. Statistical significance compares results for the EV stimulated sample to those of the A55 stimulated sample. (D). HEK293T pCW57 stable cell lines inducibly expressing C6, B14, or A55 were induced with 2 µg/ml doxycycline for 24 h, starved for 6 h in DMEM with no supplements, and left unstimulated or stimulated with TNFΑ for 18 h. Levels of secreted CXCL10 in the cell culture medium were assayed by ELISA. Data shown are representative of two independent experiments carried out in triplicate. Statistical significance compares results for C6 stimulated cells with those for B14 or A55. (E) HEK293T-REx cell lines inducibly expressing B14 or A55 were induced with 2 µg/ml doxycycline for 22.5 h and left unstimulated or stimulated with 20 ng/ml TNFΑ for 1.5 h in DMEM with no supplements. IL-8 expression levels were analyzed by RT-qPCR relative to those of GAPDH. Data presented are representative of two independent experiments carried out in triplicate. Statistical significance compares the results for C6 stimulated and A55 stimulated cells. (F and G). A55 does not inhibit mitogen-activated protein kinase (MAPK) or JAK-STAT signaling. HEK293T cells were transfected with pAP-1-Luc or pISRE-Luc, as indicated, and pRL-TK (see Materials and Methods) and plasmids expressing Flag-tagged KLHL12, C6, or A55 or empty vector (EV). After 24 h cells were stimulated with 10 ng/ml PMA (F) or 1,000 U/ml IFN-α (G) for 6 h. Cell lysates were prepared, and the fold increase in luciferase activity relative to *Renilla* activity was determined. In parallel, cell lysates were analyzed by SDS-PAGE and immunoblotting with anti-Flag or anti-α-tubulin to determine protein expression levels from unstimulated samples. Data are representative of three independent experiments. Statistical significance compares results with EV stimulated samples to those with the test sample. NS, not stimulated; IB, immunoblotting. *, *P* < 0.05; **, *P* < 0.01; ***, *P* < 0.001; ****, *P* < 0.0001.

Due to the conservation between EVM150 and A55 and because other NF-κB inhibitors exist in the VACV genome (12), a role for A55 in NF-κB signaling was investigated. HEK293T cells were cotransfected with a plasmid expressing A55 and plasmids encoding NF-κB–luciferase (Luc) and pTK-*Renilla*. Cells were stimulated with tumor necrosis factor alpha (TNF-α) or IL-1β or left unstimulated, and firefly luciferase was measured alongside *Renilla* luciferase as an internal control. Empty vector (EV) and the human BBK KLHL12 were used as negative controls, while B14 was included as a known NF-κB inhibitor. A55 expression inhibited NF-κB activity in response to both IL-1β and TNF-α compared to the activity with the EV and KLHL12 controls (Fig. 1A and B) in a dose-dependent manner (Fig. 1C). A55 also inhibited expression of endogenous NF-κB-responsive genes in response to TNF-α stimulation. For instance, transcription of IL-8 (measured by reverse transcription-quantitative PCR [RT-qPCR]) and secretion of CXCL10 (measured by enzyme-linked immunosorbent assay [ELISA]) were both inhibited by A55 (Fig. 1D and E). In contrast, A55 did not inhibit the JAK-STAT (interferon-stimulated response element [ISRE]-luc) or activator protein 1 (AP-1) promoter activity in response to alpha interferon (IFN-α) or phorbol myristic acid (PMA), respectively (Fig. 1F and G). VACV protein C6 inhibited IFN-α-stimulated ISRE activity as reported previously (Fig. 1G) (26). The ability of A55 to inhibit both IL-1β- and TNF-α-induced stimulation of NF-κB signaling suggested that it acts at or below TAK1 phosphorylation where the IL-1R and TNFR pathways converge.

### Inhibitory activity of A55 is mapped to NF-κB activation downstream to IκBα degradation.

**FIG 2:**
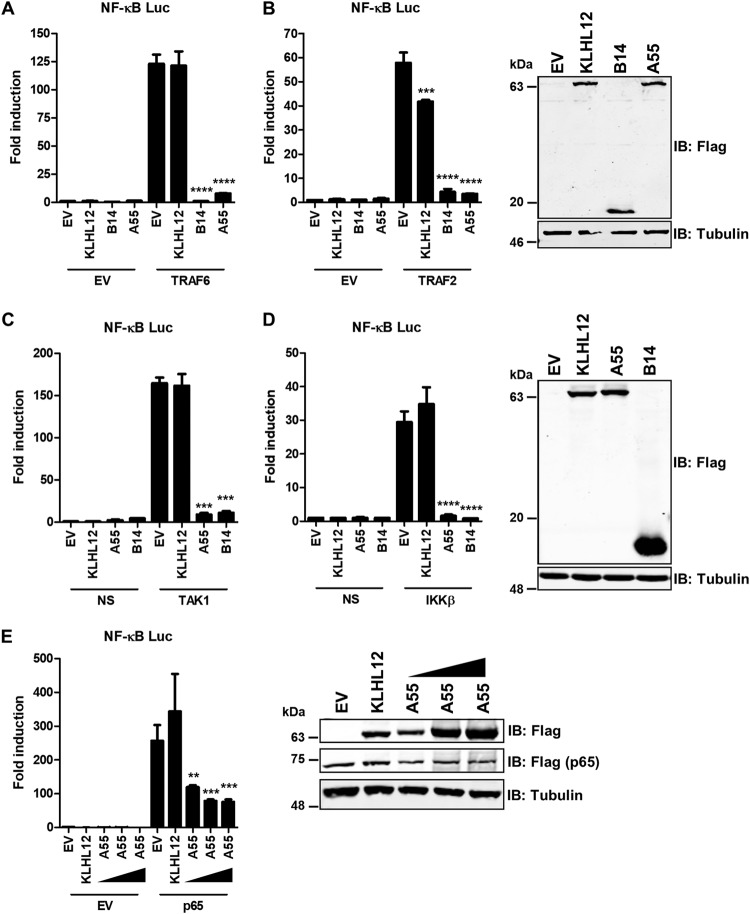
A55 inhibits NF-κB activation downstream of p65 release. (A to D) HEK293T cells were transfected with pLuc-NF-κB and pRL-TK together with plasmids expressing Flag-tagged KLHL12, B14, or A55 or EV and either TRAF6, TRAF2, TAK1, or IKKβ, as indicated. After 24 h the fold difference in luciferase activity was determined, and the abundance of indicated proteins was measured by SDS-PAGE and immunoblotting. (E) Cells were transfected with reporters as described above together with a Flag-tagged p65 expression plasmid and increasing doses of plasmid expressing A55 (25, 75, and 150 ng). Experiments are representative of three independent experiments carried out in quadruplicate. Statistical significance compares results with EV stimulated samples to those with the stimulated test sample. **, *P* < 0.01; ***, *P* < 0.001; ****, *P* < 0.0001.

The NF-κB pathway can be activated by overexpression of proteins acting at specific stages. Thus, to map where A55 inhibits the pathway, a plasmid encoding TRAF6, TRAF2, TAK1/TAB1, IKKβ, or p65 was cotransfected into HEK293T cells along with pcDNA4/TO-nTAP-coA55R. Pathway activation was measured by NF-κB-luciferase expression as described above. EV and KLHL12 or B14 were used as negative and positive controls, respectively. B14 inhibited at IKKβ (9) (Fig. 2A to D), while KLHL12 was not inhibitory (Fig. 2A to E). A55 expression inhibited NF-κB activity in response to TRAF2, TRAF6, TAK1, IKKβ, and p65 (Fig. 2A to E), and with respect to p65 the degree of inhibition increased with greater expression of A55 (Fig. 2E). Therefore, A55 acts at the level of p65 or downstream.

**FIG 3:**
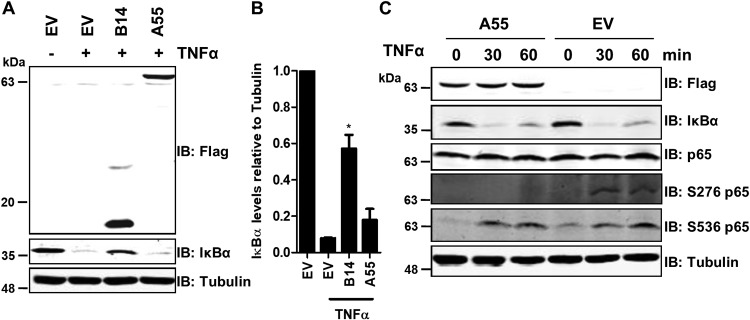
A55 acts downstream of IκBα degradation and prevents phosphorylation of p65 on Ser276. HEK293T-REx cell lines inducibly expressing B14 or A55 or empty vector (EV) were induced with 2 µg/ml doxycycline for 24 h and then stimulated with 40 ng/ml TNF-α for 20 min. The levels of IκBα, Flag-tagged B14 or A55, and α-tubulin were determined by SDS-PAGE and immunoblotting. (B) The level of IκBα relative to that of tubulin was quantified by densitometry from three independent experiments. Statistical significance compares results of the EV stimulated condition with those of the B14 or A55 stimulated condition. *, *P* < 0.05. (C). HEK293T-REx cell lines inducibly expressing A55 or EV were induced with doxycycline as described for panel A and then left unstimulated (0) or stimulated with 40 ng/ml TNF-α for 30 or 60 min. The levels of indicated proteins and phosphorylation of p65 were determined by SDS-PAGE and immunoblotting. Loading and p65 levels were controlled for using anti-tubulin and anti-p65, respectively. The immunoblot shown is representative of three independent experiments.

Under resting conditions, IκBα tethers NF-κB in the cytoplasm, but upon stimulation IκBα is phosphorylated by IKKβ, ubiquitylated, and degraded by the β-TrCP/cullin-1 E3 ligase complex and proteosome (27). Free p65 phosphorylated at S536 (p-p65 S536) translocates into the nucleus where it undergoes phosphorylation at S276. This facilitates recruitment of phosphorylated RNA polymerase II (p-RNAP II) and p300 (7) and formation of the enhanceosome to initiate gene transcription. To map the inhibitory mechanism of A55 further, HEK293T-REx cell lines with EV or vectors expressing B14 or A55 were stimulated with TNF-α, and IκBα levels were analyzed. As expected, B14 inhibited TNF-α-induced degradation of IκBα (Fig. 3A and B). However, although expression levels of A55 and B14 were similar, A55 did not prevent IκBα degradation (Fig. 3A and B). Consistent with reporter gene assays, this indicated that A55 acted at or below p65 release. Phosphorylation is key to the transcriptional activity of p65. To determine if A55 regulates phosphorylation of p65, HEK293T-REx cells expressing A55 were stimulated with TNF-α, and the levels of p-p65 S536 and S276 were analyzed. While p-p65 S536 levels were comparable in the presence or absence of A55, A55 abrogated S276 phosphorylation (Fig. 3C), which is a nuclear event (7). This suggests that A55 may inhibit S276 phosphorylation directly or indirectly by preventing nuclear translocation of p65.

**FIG 4:**
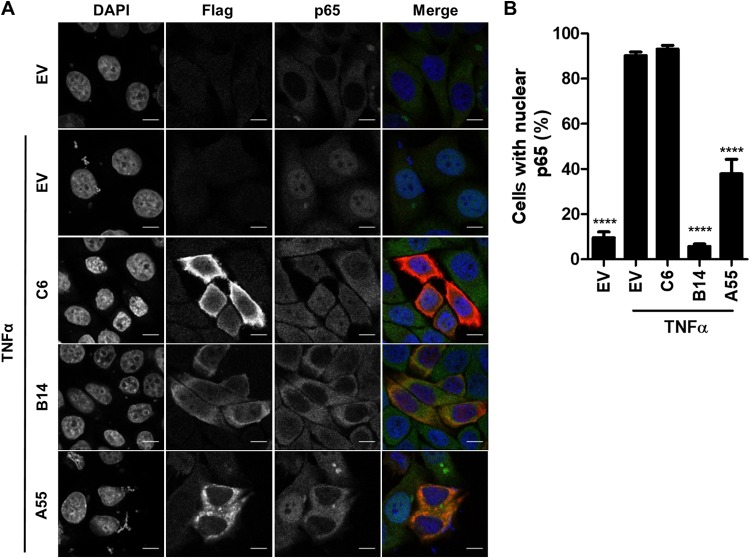
A55 inhibits p65 nuclear translocation. (A) Representative immunofluorescence staining of p65 localization in HeLa cells transfected with 1,000 ng/well of a six-well plate of plasmids encoding Flag-tagged B14, A55, C6, or EV and left untreated or stimulated with 20 ng/ml TNF-α for 30 min. Cells were stained with DAPI (blue), anti-Flag (red), and anti-p65 (green) antibodies. (B) Percentage of transfected cells with nuclear p65. Results are representative of three independent experiments carried out in triplicate, counting 100 cells per sample. Statistical significance compares the results of the EV stimulated sample with those of the A55, B14, or C6 stimulated sample. Scale bar, 10 μM. ****, *P* < 0.0001.

To explore whether A55 inhibits nuclear translocation of p65, HeLa cells transfected with Flag-tagged EV, B14, A55, or C6 were stimulated with TNF-α, and the subcellular localization of p65 was analyzed by immunofluorescence (Fig. 4A). Cells were costained with anti-Flag and anti-p65, and 4′,6′-diamidino-2-phenylindole (DAPI) was used to stain DNA; then the numbers of cells exhibiting nuclear exclusion of p65 were determined (Fig. 4B). As expected, almost 100% of the EV untreated cells exhibited nuclear exclusion of p65 in contrast to p65 exclusion in only 10% of EV-stimulated cells (Fig. 4B), and B14 prevented nuclear translocation of p65, while protein C6 did not (Fig. 4B). In comparison, A55 inhibited translocation of p65 in 62% of transfected cells following stimulation (Fig. 4B). Collectively, this maps the inhibitory activity of A55 prior to nuclear translocation of free NF-κB but after IκBα degradation.

### A55 targets importins to inhibit NF-κB activation.

**FIG 5:**
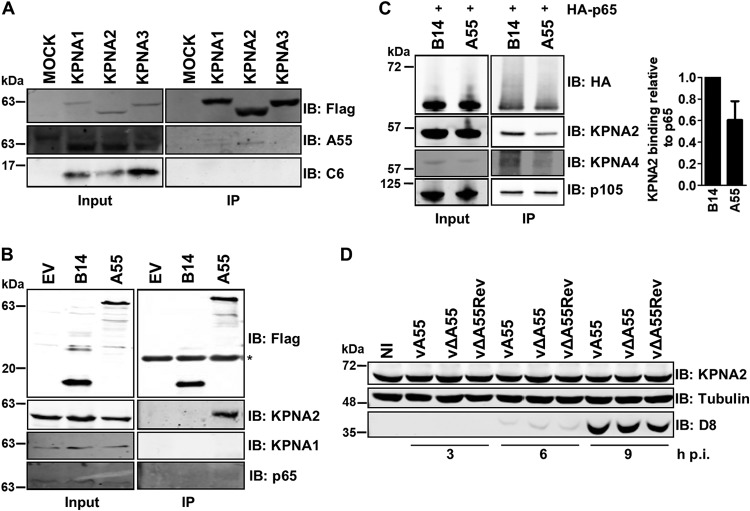
A55 targets host importin KPNA2. (A to C) Representative immunoblots showing anti-Flag or anti-HA immunoprecipitation (IP), as indicated, of cleared lysates from HEK293T cells lysed in NP-40 (A and B) or RIPA (C) buffer and subjected to SDS-PAGE and immunoblotting with the indicated antibodies. For the experiment shown in panel A, HEK293T cells were transfected with plasmids encoding Flag-KPNA1, KPNA2, KPNA3, or EV, and 24 h later cells were infected with WT VACV at 5 PFU/cell for 18 h. For the experiment shown in panel B, HEK293T-REx cells inducibly expressing B14, A55, or EV were induced with 2 µg/ml doxycycline for 36 h. For the experiment shown in panel C, pCW57 cell lines expressing B14 or A55 inducibly were induced with 2 μg/ml doxycycline for 24 h and then transfected with a plasmid encoding HA-tagged p65. Input, cleared cell lysate; IP, immunoprecipitate; IB, immunoblot. In the right panel, the amount of KPNA2 coprecipitating with p65 in the presence of A55 or B14 was quantified by densitometry. Data are the average of three independent experiments. The asterisk in panel B indicates the immunoglobulin light chain. (D) Endogenous KPNA2 levels in cell lysates from HeLa cells infected for 3, 6, or 9 h with vA55, vΔA55, or vΔA55Rev at 5 PFU/cell or left noninfected (NI). Tubulin was used as a loading control, and VACV protein D8 was used as a control for infection. The immunoblot shown is representative of two independent experiments.

The nuclear translocation of NF-κB p65 is regulated by importin-α1 to -α4/importin-β shuttling, dependent upon cell type and stimulus (3–5). To determine if A55 targets the nuclear importins, the interaction of A55 and importins was investigated following immunoprecipitation (IP) of Flag-tagged KPNA1, -2, or -3 from transfected HEK293T cells that were infected with wild-type (WT) VACV WR. Coimmunoprecipitation (co-IP) of A55 was tested using an A55-specific antibody (23). A55 coprecipitated with KPNA2, but not KPNA1 or -3, whereas C6 was not coprecipitated (Fig. 5A). Consistent with this, endogenous KPNA2 but not KPNA1 coprecipitated with transfected Flag-tagged A55 (Fig. 5B). KPNA2 is required for p65 translocation, and therefore the interaction of p65 and KPNA2 was investigated in the presence of A55 or B14. Transfected hemagglutinin (HA)-p65 was immunoprecipitated, and the levels of coprecipitated KPNA2 were reduced in the presence of A55 (Fig. 5C), suggesting that A55 may block interaction of KPNA2 and p65. As A55 is a predicted BBK E3 ligase adaptor, the levels of KPNA2 following infection with the WT VACV WR strain expressing A55 (vA55), a mutant lacking the *A55R* gene (vΔA55), or a revertant virus with *A55R* inserted back into vΔA55 and under its endogenous promoter (vΔA55Rev) were investigated. Notably, the levels of KPNA2 did not alter during infection or transfection (Fig. 5D).

### A55 inhibits NF-κB in a Kelch-dependent manner independent of cullin-3 binding.

**FIG 6:**
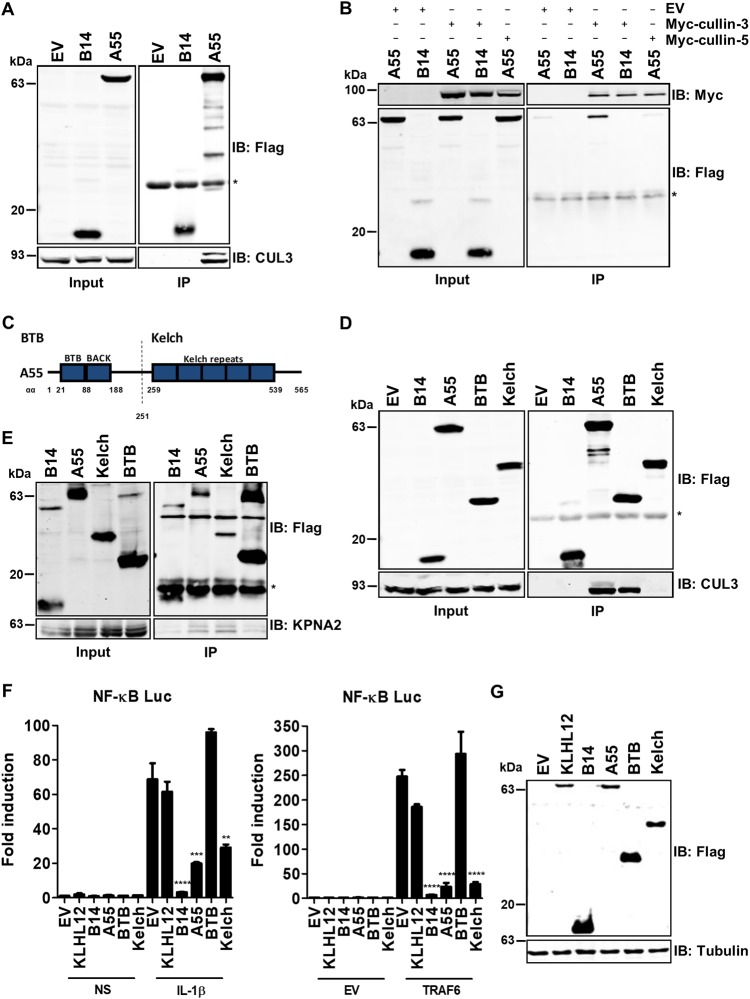
A55 inhibits NF-κB activation in a cullin-3-independent manner via its Kelch domain. Shown are immunoblots following immunoprecipitation (IP) of cleared cell lysates from HEK293T-REx cells inducibly expressing B14, A55, or EV (A), B14 or A55 (B), or B14, A55, A55-BTB, A55-Kelch, or EV (D) at 24 h postinduction with 2 μg/ml doxycycline and lysis in NP-40 (A and D) or RIPA (B) lysis buffer. Samples were subjected to SDS-PAGE and immunoblotting with the stated antibodies. (A and D) Flag-tagged immunoprecipitation and immunoblotting for endogenous cullin-3 (CUL3). (B) Reciprocal IP with protein G-Sepharose supplemented with mouse anti-Myc using cell lysates prepared 24 h posttransfection with pCDNA-Myc-*CUL3* or -*CUL5*. (C) Schematic of A55 domains. Full-length A55 from amino acid 1 to 565 was divided into the N-terminal BTB-BACK-containing domain and the C-terminal Kelch domain as depicted. (E) Flag IP as described for panel D in RIPA buffer using the pCW57 HEK cell lines expressing B14, A55, A55-BTB, or A55-Kelch and blotting for endogenous KPNA2. Input, cleared lysate; IP, immunoprecipitate; IB, immunoblot; *, antibody heavy/light chain. (F) HEK293T cells were transfected with pLuc-NF-κB and pRL-TK together with 100 ng of pcDNA3-Flag-KLHL12, 20 ng of pcDNA4-coB14R-Flag, of 100 ng of pcDNA4/TO-nTAP-coA55R, pcDNA4/TO-nTAP-coA55R-BTB, pcDNA4/TO-nTAP-coA55R-Kelch, or pcDNA4/TO-EV. In the experiment shown in the right panel, cells were also transfected with plasmid expressing TRAF6. After 24 h cells were either left unstimulated or stimulated with 15 ng/ml IL-1β for 6 h, and the luciferase and *Renilla* activities were measured. Statistical significance compared results with EV (stimulated) to those with the test samples. (G). Lysates from cells treated (as described for the left panel of F) were analyzed by SDS-PAGE and immunoblotting with the indicated antibodies. Data shown in all panels are representative of three independent experiments. **, *P* < 0.01; ***, *P* < 0.001; ****, *P* < 0.0001.

**FIG 7:**
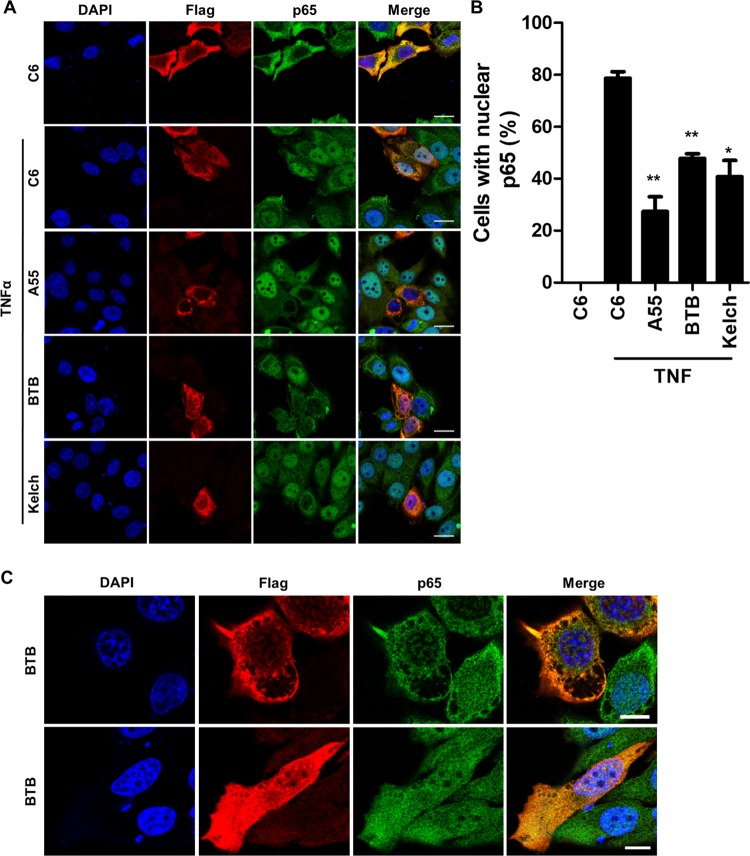
The A55 Kelch domain inhibits p65 nuclear translocation. (A) Representative immunofluorescent staining of p65 localization in HeLa cells transfected with plasmids encoding Flag-tagged B14, A55, C6, or EV and left untreated or stimulated with 40 ng/ml TNF-α for 30 min. Cells were stained as described in Materials and Methods with DAPI (blue), anti-Flag (red), and anti-p65 (green). Scale bar, 20 μM. (B) Percentage of transfected cells with nuclear p65. Data are representative of three independent experiments. Statistical significance compares results of EV (stimulated) with those of A55, B14, or C6 (stimulated). (C) Representative image of HeLa cells transfected with a plasmid encoding A55-BTB. Scale bar, 10 μM. Data are representative of three independent experiments. *, *P* < 0.05; **, *P* < 0.01.

Typically, BBKs recruit cullin-3 via their BTB domains and recruit their substrates to the cullin-3 E3 ligase complex via their C-terminal Kelch domains. Therefore, a possible interaction between A55 and cullin-3 was investigated, and this showed that endogenous cullin-3 coprecipitated with A55 but not with B14 (Fig. 6A). This interaction was confirmed by reciprocal IP of overexpressed Myc-tagged cullin-3 or cullin-5 (Fig. 6B). Subdividing A55 into its BTB and Kelch domains (Fig. 6C) showed that precipitation of endogenous cullin-3 was achieved by the BTB domain but not the Kelch domain of A55 (Fig. 6D). In contrast, the Kelch domain was sufficient to co-IP endogenous KPNA2 (Fig. 6E). To determine if the Kelch domain mediated inhibition of NF-κB activation, HEK293T-REx cells expressing the full-length A55 or the BTB-BACK or Kelch domain separately were stimulated with IL-1β or TRAF6 cotransfection, and NF-κB luciferase activity was measured. Despite higher levels of BTB-BACK expression (Fig. 6G), only the Kelch domain inhibited IL-1β- and TRAF6-induced NF-κB activation (Fig. 6F). We next tested if either domain was sufficient to prevent NF-κB p65 nuclear translocation. HeLa cells transfected with Flag-tagged C6, A55, A55 BTB, or A55 Kelch were stimulated with TNF-α, and the subcellular localization of p65 was analyzed by immunofluorescence as before. Interestingly, both domains alone appeared to inhibit p65 translocation following TNF-α stimulation as efficiently as full-length A55 (Fig. 7A and B). However, the inhibitory activity of A55 BTB-BACK correlated with induction of cellular vacuolization (Fig. 7C), compromising the conclusion that this was indirect inhibition by BTB-BACK. Thus, A55 can inhibit NF-κB activation in a Kelch-dependent and cullin-3-independent manner.

### A55 inhibits CD8^+^ T-cell proliferation and activation in the acute phase of infection.

The potential role of A55 as an immunomodulator was investigated next by measuring the immune cell number and activation status, as well as immunological memory, in C57BL/6 mice infected intradermally with vA55, vΔA55, and vΔA55Rev (28, 29).

**FIG 8:**
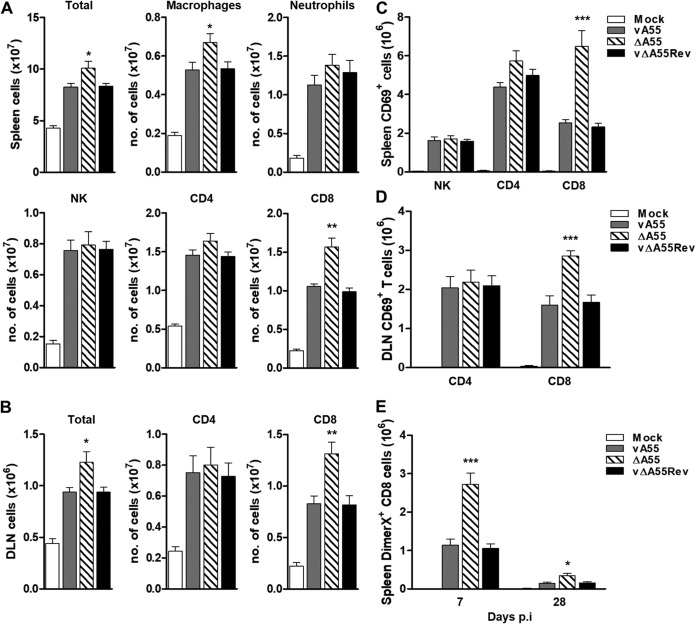
Infection with vΔA55 induces enhanced CD8^+^ T-cell response. (A and B) Female C57BL/6 mice (6 to 8 weeks old; *n* = 5) were injected intradermally in the ear pinnae with 5 × 10^3^ PFU of purified vA55, vΔA55, or vΔA55Rev or with PBS. At 7 days p.i. the total numbers of cells in spleen and draining lymph node (DLN) were determined. The absolute numbers of CD8^+^ or CD4^+^ T cells from spleen or DLN and the number of splenic NK cells, macrophages, and neutrophils were determined by fluorescence-activated cell sorting. (C and D) The expression levels of CD69 on splenic NK, CD4^+^, and CD8^+^ cells and on DLN CD4^+^ and CD8^+^ cells were quantified. (E) Number of splenic, VACV-specific, DimerX-positive CD8^+^ T cells at days 7 and 28 p.i. Statistical analysis compares results with vΔA55 to those with vA55 or vA55Rev. Data shown are representative of two independent experiments. *, *P* < 0.05; **, *P* < 0.01; ***, *P* < 0.001.

As expected, the lesion size was increased upon infection with vΔA55 compared to lesions in control virus infections (data not shown) (23). On day 7 postinfection (p.i.), the absolute cell numbers of immune cells in the spleen (Fig. 8A) and draining lymph nodes (DLN) (Fig. 8B) were counted. The total splenic and DLN cell number and the numbers of CD8^+^ T cells and macrophages in these organs were significantly increased during infection with vΔA55 compared to levels with control virus infection, while no significant changes were observed for CD4^+^ T cells, neutrophils, or NK cells (Fig. 8A and B). CD8^+^ T cells also showed enhanced activation (increased CD69 expression) at day 7 p.i. (Fig. 8C and D), and this correlated with increased VACV-specific CD8^+^ T cells at days 7 and 28 p.i. (Fig. 8E). In contrast, no increase in NK cell or CD4^+^ T-cell activity was observed (Fig. 8C and D). Interestingly, the increase in CD8^+^ T-cell activation upon infection with vΔA55 was still apparent at day 28 p.i. (Fig. 8C to E), whereas T-cell activation in the WT infection had returned to basal level. Therefore, we tested if loss of A55 enhanced CD8^+^ T-cell memory and, if so, if immunization with vΔA55 provides better protection to challenge.

### A55 deletion enhances CD8^+^ T-cell memory and provides protection to VACV challenge.

**FIG 9:**
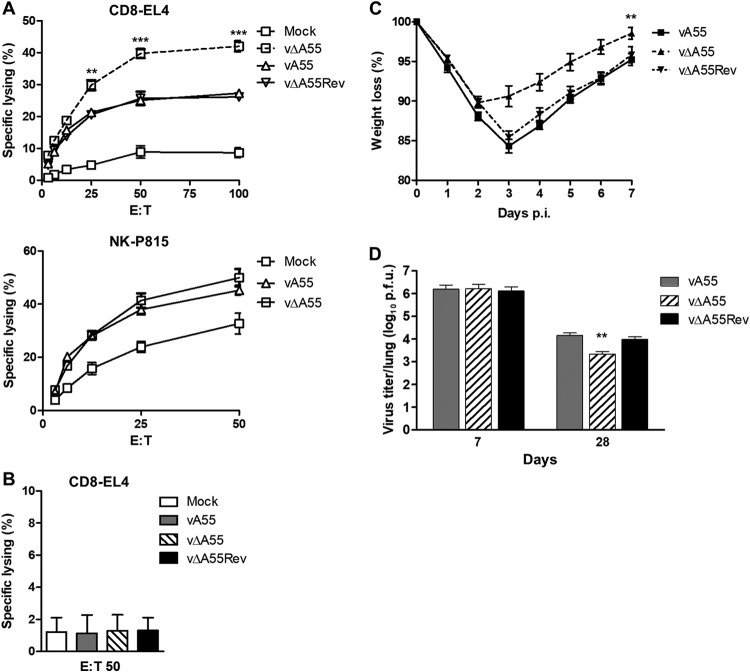
Infection with vΔA55 induces enhanced CD8^+^ T cell cytotoxicity and better protection to VACV challenge. Mice were infected as described in the legend of Fig. 7, and the immune response was analyzed at 28 days p.i. (A) ^51^Cr release assay assessing the ability of splenic CD8^+^ T cells to lyse VACV-infected EL4 cells or of splenic NK cells to lyse VACV-infected P815 cells. Data are presented as the percentage of cell lysis at various effector-to-target (E/T) cell ratios. (B) ^51^Cr release assay conducted as described for panel A but with cells preincubated with a monoclonal anti-CD8 antibody. (C and D) At 28 days p.i. mice immunized with vA55, vΔA55, or vΔA55Rev were infected with 10^7^ PFU of WT VACV, and weight change and pulmonary virus titers were determined. Statistical analysis compares results with vΔA55 to those with vA55 or vA55Rev. Data shown are representative of two independent experiments. **, *P* < 0.01; ***, *P* < 0.001.

Strong CD8^+^ T-cell immunological memory is desirable following vaccination (30). To test if A55 modulates immunological memory, the killing activity of splenic CD8^+^ T cells and NK cells was assessed at day 28 p.i. with vA55, vΔA55, or vΔA55Rev. Killing of VACV-infected EL4 cells by splenic T cells isolated 28 days p.i. was significantly increased for all three infected groups versus levels for the mock-infected control (Fig. 9A). EL4 killing was also greater with CD8^+^ T cells isolated from mice immunized with vΔA55 than with those from mice immunized with control viruses (Fig. 9A). Notably T-cell-mediated killing was reduced to basal levels by the addition of an anti-CD8 antibody, showing that cytolysis was CD8^+^ T cell specific (Fig. 9B). In contrast, no differences between the vA55 or vΔA55 groups were observed in the ability of NK cells to kill VACV-infected P815 cells following immunization (Fig. 9A).

To test if vΔA55 would be a better vaccine, mice infected intradermally with vA55, vΔA55, or vA55Rev were challenged intranasally at day 28 p.i. with vA55. Weight loss and viral titers in the lungs were quantified on days 1 and 4 p.i. Weight loss was significantly reduced from day 3 to day 7 p.i. in vΔA55-immunized mice compared to levels in the control groups (Fig. 9C). This was accompanied by a decrease in viral titer in the lungs of vΔA55-immunized mice at day 4 p.i. (Fig. 9D). Taken together, these data show that vΔA55-immunized mice are better protected following intranasal challenge and clear viral infection more quickly, making vΔA55 a more efficacious vaccine.

## DISCUSSION

During evolution with their hosts, viruses have evolved strategies to evade or suppress the host immune system. The study of virus-encoded proteins that modulate the immune response (immunomodulators or immunoevasins) has increased our understanding of not only virus immune evasion and pathogenesis but also cellular signaling pathways and regulators of the immune response to infection. VACV encodes scores of immunomodulators, and a mutant virus lacking one of these, called A55, increased immunopathology following intradermal infection (23). This observation and the report that the orthologue of A55 in ectromelia virus, EVM150, inhibited NF-κB (13) led us to investigate the function of A55 further and the consequence of A55 expression on the NF-κB signaling pathway *in vitro* and on the immune response to infection *in vivo*. This report shows that A55 targets the cellular importin KPNA2 to dysregulate p65 translocation and restrict NF-κB activation. Furthermore, immunization with a virus lacking A55 induced a stronger CD8^+^ T-cell response to VACV and better protection against VACV challenge.

Consistent with a report on EVM150 (13), data presented show that A55 inhibits cytokine expression and NF-κB-luciferase activity in response to TNF-α and IL-1β by preventing p65 translocation and without affecting IκBα levels. This suggested a possible modulation of cellular factors needed for p65 translocation. Consistent with this, A55 coprecipitated with KPNA2, part of the importin complex. Further, the interaction seen between KPNA2 and p65 was reduced in the presence of A55, indicating that A55 may disrupt KPNA2-p65 complex formation to inhibit p65 translocation. This observation is relevant to understanding which KPNAs control NF-κB p65 translocation.

Humans encode a family of importins among which KPNA1 to -4 and KPNA6 are implicated in regulating NF-κB signaling. The interaction of KPNA1 to -3 with NF-κB was identified from an *in vitro* screen using importins purified from bacteria (5), and an interaction of KPNA2 to -4 with p65 was observed both *in vitro* and *in vivo*; the KPNA2-p65 interaction was also nuclear localization signal (NLS) dependent (4). Conversely, KPNA2-p65 binding was not observed using recombinant protein from insect cells, and the authors suggested that interaction with KPNA3 and -4 (KPNA3/4) is more biologically relevant (4). However, this evidence may only suggest that KPNA2 does not interact directly with p65 in the absence of pathway stimulation or posttranslation modification (4) and that KPNA2-p65 binding was enhanced following TNF-α stimulation (4). A small interfering RNA (siRNA) screen of the impact of importins on p65 translocation following TNF-α stimulation indicated that KPNA2 and KPNA6 contribute the most (3). More recently siRNA-mediated knockdown of KPNA2 was shown to impair p65 translocation and NF-κB-dependent gene expression in rat pancreatic acinar cells (6). Collectively, the literature suggests that multiple importins regulate p65 translocation, with KPNA2 playing a leading role. KPNA redundancy may explain why A55-mediated inhibition of p65 translocation is incomplete and why its inhibition is less efficient than that of B14, which targets IKKβ (9), or that of A49, which targets β-TrCP (11).

Targeting of importins to dysregulate cellular signaling has been reported for RNA viruses (31–34). For instance, Japanese encephalitis virus NS5 targets KPNA2 to -4, with binding of KPNA3/4, preventing IRF3 translocation (32). Hepatitis C virus NS3/4A restricts IRF3 and NF-κB translocation by targeting KPNAB1 (33), and the Hantaan virus nucleocapsid protein inhibits p65 translocation and coprecipitated with overexpressed KPNA1, -2, and -4 (34). However, this report is the first describing specific targeting of KPNA2 to disrupt p65-KPNA2 complex formation and subsequently NF-κB signaling and reinforces the importance of KPNA2 in NF-κB translocation. A55 lacks a canonical NLS and is cytoplasmic; it is therefore unlikely to be a KPNA2-dependent cargo but, rather, functions in the cytoplasm. While targeting KPNA2 reduces NF-κB signaling, it may also have other consequences on nuclear shuttling that may contribute to the *in vivo* phenotype of vΔA55. The A55 N-terminal BTB domain, which binds cullin-3, may also contribute to this phenotype.

Although the inhibition of NF-κB activation by A55 depends on the A55 Kelch domain and is independent of cullin-3 binding, the BTB-BACK domain likely also has an important function. To start to address this, the cocrystal structure of the A55 BTB-BACK domain bound to cullin-3 was determined and revealed an overall interaction that is conserved between cullin-3 and other cellular binding partners such as KLHL3, a human BTB (42). Nonetheless, the affinity of A55 BTB-BACK for cullin-3 was much higher than for the interaction of cullin-3 with other cullin-3 binding partners, suggesting that A55 would be an effective competitor for the interaction of cullin-3 and its binding partners (42).

Data presented here are consistent with those for the ectromelia virus orthologue of A55, EVM150, with one exception. Both proteins coprecipitated with cullin-3 via an N-terminal BTB domain, and both inhibited NF-κB activation in a cullin-3-independent manner. With EVM150, however, inhibition of p65 translocation and of NF-κB signaling was reported to require only the BTB domain, and the Kelch domain lacked this inhibitory activity (13). In contrast, the A55 Kelch domain mediated coprecipitation with KPNA2 and inhibition of NF-κB activation, and this fits with inhibition being cullin-3 independent. Discrepancies might reflect precisely where A55 and EVM150 were subdivided, subtle differences in amino acid composition, or changes in solubility and expression levels of the BTB and Kelch domains. Concerning these possibilities, first, we expressed the A55 BTB domain as amino acids 1 to 251, a region which includes the BACK and 3-box domains. The Kelch domain was expressed as amino acids 252 to 565. In contrast, the study of EVM150 expressed the BTB domain only, lacking both the BACK and 3-box domains. Second, we observed that the A55 BTB-BACK domain expressed at higher levels than either the Kelch domain or full-length protein, and this was adjusted for the reporter assays by modulating the amount of plasmid transfected. Finally, expression of A55 BTB-BACK induced cellular vacuolization, a phenotype associated with cullin-3 depletion (35). Therefore, the apparent inhibitory activity of A55 BTB-BACK and potentially of EVM150-BTB may be due to overexpression and cullin-3 saturation leading to inhibition of p65 translocation due to loss of cell viability. While the Kelch domain alone was sufficient to inhibit NF-κB signaling, the ability of A55 to bind cullin-3 might still affect NF-κB signaling during infection, when protein levels may be lower and other viral proteins are present, and particularly *in vivo*, where NF-κB activation needs to be counteracted in different cell types. The existence and conservation of several poxviral BBKs suggest that they play an important role during infection.

Inflammation and expression of NF-κB-dependent cytokines are important for the development and proliferation of effector and memory T-cell populations (36). Consistent with this, deletion of the NF-κB inhibitor A55 modulated the immune response during acute infection and led to enhanced VACV-specific CD8^+^ T-cell memory. This is reminiscent of a VACV mutant lacking NF-κB inhibitor N1 causing enhanced CD8^+^ T-cell memory and effector function (37). However, to confirm that it is the ability of A55 to inhibit NF-κB activation that results in the enhanced CD8^+^ T-cell memory, rather than other functions of the A55 protein, it will be necessary in the future to study the immunogenicity of a virus expressing a mutant A55 protein that is no longer able to bind to cullin-3. Understanding the signals derived from acute infection that mediate development of immunological memory is key to effective future vaccine design. VACV mutants lacking specific immunomodulators are useful tools to understand the mechanisms underlying and controlling immunological memory but also suggest ways to produce VACV-based vaccines that induce enhanced CD8^+^ T-cell memory.

In conclusion, this report shows that VACV protein A55 inhibits NF-κB activation and is the 11th VACV-encoded NF-κB inhibitor (8). Despite the presence of other NF-κB inhibitors, loss of A55 gives an *in vivo* phenotype leading to enhanced CD8^+^ T-cell memory and better protection against reinfection. Mechanistically, A55 coprecipitates with KPNA2 via its Kelch domain to disrupt p65-KPNA2 interaction and impair p65 nuclear translocation.

## MATERIALS AND METHODS

### Ethics statement.

This work was conducted under license PPL 70/8524 from the UK Home Office according to the Animals (Scientific Procedures) Act 1986.

### Cells, plasmids, reagents, and viruses.

All reagents were purchased from Sigma unless otherwise stated. BSC-1 (ATCC) and HEK293T cells were grown in high-glucose Dulbecco’s modified Eagle’s medium (DMEM) (Gibco), HeLa cells (ATCC) were grown in minimal essential medium (MEM; Gibco), and EL4 (H-2b; ATCC) and P815 (ATCC) cells were grown in RPMI medium (Gibco). All media were supplemented with 10% fetal bovine serum (FBS; Pan Biotech), nonessential amino acids (NEAA), and 50 μg/ml penicillin-streptomycin (P-S) at 37°C in a 5% CO_2_ atmosphere. All plasmids used and those constructed during this study are listed in Table 1. Plasmids were generated using conventional restriction enzyme digest and ligation using the primers with restriction enzyme sites indicated in Table 1. VACV strain WR recombinants vA55 (plaque purified wild-type), vΔA55 (A55 deletion mutant), and vΔA55Rev (revertant virus with A55R reinserted into vΔA55) were described previously (23). Infectious virus titers (number of PFU/milliliter) were determined by plaque assay on BSC-1 cells.

**TABLE 1:** Plasmids constructed or used in this study

Constructed plasmid^*a*^	Description or primer sequence^*b*^	Restriction enzyme	Reference or source
**pcDNA4/TO-nTAP/nHA/cTAP**	Mammalian expression vector with N-terminal or C-terminal Strep-I and Strep-II tag followed by Flag tag or with a C-terminal HA tag only or a C-terminal Flag tag only, all under the control of a CMV promoter; Amp^r^, Zeo^r^		Invitrogen
pcDNA4/TO-nTAP-*A55R*	GACGCGGCCGCGAACAACAGCAGCGAGC	NotI	This study
	GACTCTAGATCAGCTTCCGATGAAGC	XbaI	
pcDNA4/TO-nTAP-*A55R* BTB	GACGCGGCCGCGAACAACAGCAGCGAGC	NotI	This study
	GACTCTAGATTAGTGGTATCTGGGGAAGC	XbaI	
pcDNA4/TO-nTAP-*A55R* Kelch	GACGCGGCCGCGTCCATCGAGCTGATCAGC	NotI	This study
	GACTCTAGATCAGCTTCCGATGAAGC	XbaI	
pcDNA4/TO-*P65*-cTAP	ATATCGGATCCGCCACCATGGACGAACTGTTCCCCCTCATCT	BamHI	This study
	ATACTGCGGCCGCGGAGCTGATCTGACTCAGC	NotI	
pcDNA4/TO-*P65*-HA	ATATCGCGGCCGCGGACGAACTGTTCCCCCTCATCT	NotI	This study
	ATACTTCTAGATTAGGAGCTGATCTGACTCAGC	XbaI	
pCDNA4/TO-*B14R*-cTAP	GCGCGGATCCACCATGACGGCCAACTTTAGTACCCACGTCGCTGCCTCCTCCCTTTTCAAACTGAGGATGAGACCACGCGGCCGCATTCATACGCCGGAATATGGCCGCTGGCTCCCTTCTCGAACTGGGGGTGGCTCCAGCTTCCGCCTCCGCTGCCTCCTCCCTT	BamHI	This study
	CGGCTCTAGACCGCGGTTACTTGTCGTCATCGTCATCCTTGTAGTCCTCGCCGCTGGCTCCCTTCTC	XbaI	
**pCDNA6/TR**	Mammalian expression vector with the Tet repressor under the control of a CMV promoter; Amp^r^, Blast^r^		Invitrogen
**pCDNA3-Flag/HA/Myc**	Mammalian expression vector with Flag or HA tag under the control of a CMV promoter; Amp^r^		Invitrogen
pcDNA3-HA-*TAK1*	GCTGCGGCCGCTTCTACAGCCTCTGCC	NotI	This study
	GTCTCTAGATCATGAAGTGCCTTGTCG	XbaI	
pcDNA3-HA-*TAB1*	GCTGCGGCCGCATGGCGGCGCAGAGGAGGAGC	NotI	This study
	GTCTCTAGACTACGGTGCTGTCACCACGCT	XbaI	
pcDNA3-Flag-*KLHL12*			Gift from Randall T. Moon
pcDNA3-Flag-*CUL3*			Addgene, 19893
pcDNA3-Flag-*CUL5*			Addgene, 19895
**M5P-Flag**	Mammalian expression vector with Flag tag under the control of the murine leukemia virus long terminal repeat promoter; Amp^r^		
M5P-Flag-*TRAF6*			Gift from Andrew Bowie
**pCW57-GFP-P2A-MCS**	Lentiviral expression plasmid with GFP in MSC1 and P2A skip sequence followed by MCS2 under the control of a CMV promoter; Amp^r^, Puro^r^		Addgene, 89181
pCW57-GFP-P2A-nTAP-*A55R*	CGACGCGTATGTGGTCTCATCCTCAGTTTG	MluI	This study
	GCAGGATCCTCAGCTTCCGATGAAGCTTTC	BamHI	
pCW57-GFP-P2A-nTAP-*A55R* BTB	CGACGCGTATGTGGTCTCATCCTCAGTTTG	MluI	This study
	GCAGGATCCTCAGTGGTATCTGGGGAAGC	BamHI	
pCW57-GFP-P2A-nTAP-*A55R* Kelch	CGACGCGTATGTGGTCTCATCCTCAGTTTG	MluI	This study
	GCAGGATCCTCAGCTTCCGATGAAGCTTTC	BamHI	
pCW57-GFP-P2A-nTAP-*B14R*	CGACGCGTATGTGGTCTCATCCTCAGTTTG	MluI	This study
	GCAGGATCCTCAATTCATACGCCGGAATAT	BamHI	
pCW57-GFP-P2A-nTAP-*C6L*	CGACGCGTATGTGGTCTCATCCTCAGTTTG	MluI	This study
	CGACGCGTTTATCATCTGTCCACGTCGT	MluI	
**pCMV-PACK**	Packaging plasmid for lentivirus production with HIV Gag, Pol, Rev, and Tat under the CMV promotor; Amp^r^		Gift from H. Laman
**pCMV-ENV**	VSV-G pseudotyped envelope protein under the CMV promoter for lentivirus production; Amp^r^		Gift from H. Laman
**pLDT-TetR**	Lentiviral expression plasmid constitutively expressing the tetracycline promoter repressor; Amp^r^, Neo^r^		41
**pLDT-MCS**	Lentiviral expression plasmid with expression under the control of a tetracycline-inducible CMV promoter; Amp^r^, Puro^r^		41
pLDT-nTAP-*A55R*	CTAGCTAGCATGTGGTCTCATCCTCAGTTTG	NheI	This study
	GCGGTCGACTCAGCTTCCGATGAAGCTTTC	SalI	
pLDT-nTAP-*B14R*	CTAGCTAGCATGTGGTCTCATCCTCAGTTTG	NheI	This study
	CGGAATTCTCAATTCATACGCCGGAATAT	EcoRI	
pLDT-nTAP-*C6L*	CTAGCTAGCATGTGGTCTCATCCTCAGTTTG	NheI	This study
	GAGCTCGAATTCTTATCATCTGTCCACGTCGT	EcoRI	
**pCAGGS-Flag**	Mammalian expression vector with a Flag tag under the control of the CMV immediate early promoter; Amp^r^		
pCAGGS-Flag-KPNA1			31
pCAGGS-Flag-KPNA2			31
pCAGGS-Flag-KPNA3			31

a Parental plasmids are in boldface. GFP, green fluorescent protein; MCS, multiple cloning site; CMV, cytomegalovirus.

b Restriction sites are underlined. Amp^r^, ampicillin resistance; Puro^r^, puromycin resistance; Neo^r^, neomycin resistance; Zeo^r^, zeocin resistance; Blast^r^, blasticidin resistance; VSV-G, vesicular stomatitis virus protein G.

### HEK293T-REx, pLDT, and pCW57 cell line generation.

HEK293T-REx cells were constructed using the pcDNA4/TO plasmids listed in Table 1 along with plasmid pcDNA6-TR encoding the tetracycline inducible promoter repressor. After transfection into HEK293T cells using LT1 transfection reagent, cells were selected using blasticidin and zeocin, according to the manufacturer’s instructions (Invitrogen). The pCW57 or pLDT plasmids listed in Table 1, along with the EV as a control, were cotransfected with the packaging (pCMV-PACK) and envelope (pCMV-ENV) plasmids for lentivirus production in HEK293T or HEK293T TetR (pLDT-TetR transduced HEK293T) cells, respectively, using LT1 transfection reagent (MirusBio). After 48 h the medium was filtered and transferred to fresh monolayers of HEK293T cells, and 3 days later 2 μg/ml puromycin (Invivogen) was added. Transduction and expression were confirmed by the addition of 2 μg/ml doxycycline and immunoblotting of cell lysates.

### Luciferase reporter assay.

HEK293T cells were transfected with 100 ng of pcDNA4/TO-EV, 100 ng of pcDNA4/TO-nTAP-coA55R, 25 ng of pcDNA4/TO-nTAP-coA55R-BTB, 100 ng of pcDNA4/TO-nTAP-coA55R-Kelch, 100 ng of pcDNA3-Flag-*KLHL12*, 20 ng of pcDNA3-Flag-coB14R (9) or 20 ng of pcDNA4/TO-cTAP-*C6L* (38) along with 10 ng of plasmid pTK-*Renilla* (pRL-TK; Promega) and 45 ng of pLUC-NF-κB (R. Hofmeister, University of Regensburg, Germany), pLUC-AP-1 (Andrew Bowie, Trinity College Dublin), or pLUC-ISRE (Promega) using LT1 transfection reagent (MirusBio, Ltd.). All conditions were transfected with equal amounts of total plasmid DNA by supplementation with pcDNA/TO-EV. Simultaneously, where stated, plasmids encoding TRAF2, TRAF6 (Andrew Bowie, Trinity College Dublin), TAK1, TAB1, IKKβ (Alain Chariot, University of Leige), and p65 were cotransfected. After 24 h cells were stimulated with 20 ng/ml TNF-α, 15 ng/ml IL-1β, 1,000 U of alpha interferon (IFN-α) (all Peprotech), or 10 ng/ml phorbol 12-myristate 13-acetate (PMA) for 6 h. Cells were lysed in passive lysis buffer (Promega), and firefly/*Renilla* luciferase activity was measured using a FLUOstar Omega plate reader (BMG Labtech). Relative luminescence levels were calculated by normalizing firefly luminescence to *Renilla* luminescence and are represented as relative to levels of the nonstimulated EV condition.

### RT-qPCR.

RNA extraction, cDNA synthesis, and RT-qPCR were carried out as described previously (39). qPCR used primers for IL-8 (AGAAACCACCGGAAGGAACCATCT and AGAGCTGCAGAAATCAGGAAGGCT) and glyceraldehyde-3-phosphate dehydrogenase (GAPDH) (TCGACAGTCAGCCGCATCTTCTTT and ACCAAATCCGTTGACTCCGACCTT).

### ELISAs.

HEK293T-REx cell lines were starved for 6 h in DMEM without supplements before being stimulated for 18 h with 20 ng/ml TNF-α. CXCL10 in the medium was measured using a CXCL10/IP-10 DuoSet ELISA kit (R&D Systems) and a FLUOstar Omega plate reader (BMG Labtech). Experiments were carried out in triplicate and measured with technical repeats.

### Translocation assay and immunofluorescent staining.

HeLa cells were transfected with plasmids encoding N-terminal tandem affinity purification (TAP)-tagged C6, B14, or A55 using LT1 transfection reagent (MirusBio). After 24 h cells were starved in DMEM without serum for 3 h, followed by stimulation with 40 ng/ml TNF-α for 30 min. Cells were washed three times in ice-cold phosphate-buffered saline (PBS) and processed for immunofluorescence staining and imaging as described previously (38). Rabbit anti-Flag and mouse anti-p65 were used as the primary antibodies, and goat anti-rabbit 546 and donkey anti-mouse 488 were used as the secondary antibodies. Images were analyzed using Zeiss Zen microscope software and ImageJ. Experiments were performed in triplicate and carried out three times. For each repeat, 100 Flag-positive cells were counted for each condition, and the numbers of cells showing clear nuclear exclusion of p65 were counted to calculate the percentage.

### Coimmunoprecipitation.

HEK293T-REx cells were induced with 2 µg/ml doxycycline for 24 h and lysed in either phosphate-buffered saline supplemented with 0.5% NP-40 (Igepal CA-630) and protease inhibitor or in radioimmunoprecipitation assay (RIPA) buffer (50 mM Tris, pH 8.0, 150 mM NaCl, 0.5 M EDTA, 1% NP-40, 0.5% sodium deoxycholate, 0.1% SDS supplemented with protease inhibitor) where stated. Proteins were immunoprecipitated as described previously (38) with M2 Flag-tagged beads, HA-tagged beads, or Fastflow G-Sepharose (GE Healthcare) incubated previously with mouse monoclonal anti-Myc tag clone 9B11 (catalog number 2276; Cell Signaling Technology [CST]) at 1:50. After the final wash, beads were incubated in 4× sample loading dye (lithium dodecyl sulfate [LDS]; 0.5 M Tris, pH 6.8, 40% glycerol, 6% SDS, 1% bromophenol blue, and 0.8% β-mercaptoethanol) and analyzed by immunoblotting.

### Immunoblotting and antibodies.

Samples were prepared by the addition of LDS, boiled, and then separated by electrophoresis in an SDS-polyacrylamide gel in Tris-glycine-SDS (TGS) buffer (20 mM Tris, 192 mM glycine, 1% [wt/vol] SDS) before being transferred to a nitrocellulose membrane (GE Healthcare) in Tris-glycine (TG) buffer (20 mM Tris-HCl, pH 8.3, 150 mM glycine) using the Turboblot system (BioRAD). Membranes were blocked in 5% milk in Tris-buffered saline (10 mM Tris, 150 mM NaCl), pH 7.4, with 0.1% (vol/vol) Tween 20 (TBS-T) for 60 min before being incubated with the primary antibody overnight at 4°C. Primary antibodies were rabbit polyclonal anti-KPNA2 (ab70160; Abcam), rabbit polyclonal anti-IκBα (9242; CST), anti-p65 S536 (S3010S; CST), anti-p65 S276 (sc-101749; Santa Cruz), rabbit monoclonal anti-cullin-3 clone EPR3195 (ab108407; Abcam), anti-Flag (F7425), mouse anti-myc 9B11 (2276; CST), anti-tubulin (05-829; Millipore), mouse monoclonal anti-KPNA1 187.1 (sc-101292; Santa Cruz), anti-p65 clone F-6 (sc-8008; Santa Cruz), and anti-phospho-IκBα (9246; CST); antibodies to VACV proteins were rabbit polyclonal anti-C6 (38) or A55 (23) or mouse monoclonal D8 clone AB1.1 (40). Membranes were washed three times in TBS-T before incubation with secondary antibodies for 1.5 h. Secondary antibodies were goat anti-rabbit IRDye 800CW (926-68032211; LiCOR) and goat anti-mouse IRDye 608LT (926-68020; LiCOR); for immunoprecipitated samples, biotin-anti-mouse light chain followed by streptavidin IRDye 680LT (926-68031; LiCOR) was used. Finally, membranes were washed three times in TBS-T, dried, and imaged using the LiCOR system and Odyssey software. Densitometry was calculated using ImageJ.

### Intradermal mouse model of infection and intranasal challenge experiments.

Female C57BL/6 mice 6 to 8 weeks old (*n* = 5) were infected with 5 × 10^3^ PFU in both ear pinnae with VACV strains vA55, vΔA55, and vΔA55Rev that had been purified by sucrose density gradient centrifugation. Lesion size was measured daily with a micrometer until day 21. At day 28 p.i. mice were infected intranasally with 10^7^ PFU of VACV, and their weights were measured daily for 7 days.

### Isolation of cell populations and staining for flow cytometry.

Cells from spleen and lymph nodes were isolated by grinding the organ through a 40-µm-pore-size nylon cell strainer to create single-cell suspensions. Splenocytes were treated with red blood cell (RBC) lysis buffer to remove contaminating RBCs. Single-cell suspensions were stained with fluorescently labeled antibodies: CD3 (clone 145-2C11), CD4 (GK1.5), CD8 (5H10-1), CD45R (RA-6B2), NK1.1 (PK136), CD69 (H1.2F3), F4/80 (BM8), Ly6G (1A8), and CD16/32 (2.4G2) (purchased from BD Biosciences or BioLegend). These antibodies were purified or conjugated with peridinin chlorophyll protein (PerCP)/Cy5.5, fluorescein isothiocyanate (FITC), allophycocyanin (APC)/Cy7, APC, phycoerythrin (PE)-Cy7, PE, or BV650. Isotype controls were used as negative controls. Live cells were discriminated with a fixable Live/Dead stain (Life Technologies). Stained cells were analyzed by flow cytometry on a BD LSR Fortessa (BD Biosciences), and data were analyzed with FlowJo software (Tree Star, Inc.).

### DimerX assay to detect VACV-specific CD8^+^ T cells.

A DimerX assay was performed according to the manufacturer's instructions (BD Biosciences) using H-2K^b^–Ig fusion proteins and B8_20_ peptide (TSYKFESV), as described previously (37).

### CD8^+^ T-cell and NK cell killing assay.

Cytotoxic T lymphocyte activity was assayed by ^51^Cr release assay as described previously (37). VACV-infected EL4 cells or P815 cells were used as targets for VACV-specific cytotoxic T-lymphocyte cell lysis or VACV-specific natural killer (NK) cell cytotoxicity, respectively.

### Statistics.

All experiments were carried out in triplicate and are representative or an average of at least three independent experiments unless otherwise stated. Data are the means ± standard deviations (SD) or, for *in vivo* data, ± standard errors of the means (SEM). All assays were analyzed by unpaired *t* test with GraphPad Prism, version 6, software.
